# *Schistosoma mansoni* reinfection: Analysis of risk factors by classification and regression tree (CART) modeling

**DOI:** 10.1371/journal.pone.0182197

**Published:** 2017-08-16

**Authors:** Andréa Gazzinelli, Roberta Oliveira-Prado, Leonardo Ferreira Matoso, Bráulio M. Veloso, Gisele Andrade, Helmut Kloos, Jeffrey M. Bethony, Renato M. Assunção, Rodrigo Correa-Oliveira

**Affiliations:** 1 Escola de Enfermagem, Universidade Federal de Minas Gerais, Belo Horizonte, Minas Gerais, Brazil; 2 Instituto Nacional de Ciência e Tecnologia em Doenças Tropicais (INCT-DT), Salvador, Bahia, Brazil; 3 Instituto de Ciências Exatas, Universidade Federal de Minas Gerais, Belo Horizonte, Minas Gerais, Brazil; 4 Department of Epidemiology and Biostatistics, University of California Medical Center, San Francisco, California, United States of America; 5 Microbiology, Immunology and Tropical Medicine, School of Medicine and Health Science, George Washington University, Washington DC, United States of America; 6 Centro de Pesquisas René Rachou, Fundação Oswaldo Cruz, Belo Horizonte, Minas Gerais, Brazil; Brown University, UNITED STATES

## Abstract

Praziquantel (PZQ) is an effective chemotherapy for schistosomiasis mansoni and a mainstay for its control and potential elimination. However, it does not prevent against reinfection, which can occur rapidly in areas with active transmission. A guide to ranking the risk factors for *Schistosoma mansoni* reinfection would greatly contribute to prioritizing resources and focusing prevention and control measures to prevent rapid reinfection. The objective of the current study was to explore the relationship among the socioeconomic, demographic, and epidemiological factors that can influence reinfection by *S*. *mansoni* one year after successful treatment with PZQ in school-aged children in Northeastern Minas Gerais state Brazil. Parasitological, socioeconomic, demographic, and water contact information were surveyed in 506 *S*. *mansoni*-infected individuals, aged 6 to 15 years, resident in these endemic areas. Eligible individuals were treated with PZQ until they were determined to be negative by the absence of *S*. *mansoni* eggs in the feces on two consecutive days of Kato-Katz fecal thick smear. These individuals were surveyed again 12 months from the date of successful treatment with PZQ. A classification and regression tree modeling (CART) was then used to explore the relationship between socioeconomic, demographic, and epidemiological variables and their reinfection status. The most important risk factor identified for *S*. *mansoni* reinfection was their “heavy” infection at baseline. Additional analyses, excluding heavy infection status, showed that lower socioeconomic status and a lower level of education of the household head were also most important risk factors for *S*. *mansoni* reinfection. Our results provide an important contribution toward the control and possible elimination of schistosomiasis by identifying three major risk factors that can be used for targeted treatment and monitoring of reinfection. We suggest that control measures that target heavily infected children in the most economically disadvantaged households would be most beneficial to maintain the success of mass chemotherapy campaigns.

## Introduction

Despite recent progress in controlling *Schistosoma mansoni* infection in Latin America and the Caribbean (LAC) countries, this parasitic nematode remains a major public health problem in Brazil and in other low and middle income countries in the region [1]. Brazil has the largest number of *S*. *mansoni* cases among the LAC countries with approximately 6 million people currently infected, and 25 million at a risk of infection [2]. Although *S*. *mansoni* infection occurs when individuals enter fecally contaminated water inhabited by infected intermediate hosts (*Biomphalaria glabrata*), the transmission of this neglected tropical disease also has a complex social, biological, and environmental basis that must be considered for successful control and elimination programs. While the importance of these factors in the occurrence of schistosomiasis has been intensively studied [3–5], the complexity of socioeconomic and behavioral factors can vary considerably on a local level as well as on a global scale. For example, a study conducted on the shores and islands of Lake Victoria in Nyanza Province, Kenya showed that human population density was a major factor driving *Schistosoma* transmission, with individual socioeconomic factors appearing not to significantly affect transmission [6]. On the other hand, in a study conducted in Porto de Galinhas in Northeastern Brazil, the education level of heads of families and low household income were the major risk factors influencing infection [7]. Thus, mapping the risk factors for *S*. *mansoni* transmission depends on identifying the local context in which transmission occurs, which facilitates the implementation and optimization of schistosomiasis control programs. The planning and implementation of effective schistosomiasis control programs demands consideration of the major risk factors of particular communities, including their unique ecological, social, and behavioral determinants and the interaction of these determinants within the context of the control programs [8,9]. An approach that can identify a hierarchy of risk factors and their interaction at the local level could form the basis for an effective community-based control program, as it would help prioritize both the foci and the methods for the prevention or control of transmission [10]. Many previous epidemiological studies of schistosomiasis used multinomial models, such as logistic regression, which uses linear combinations as the primary method of expressing associations between variables and outcomes such as infection or reinfection [11,12]. These models do not estimate the interaction of the explanatory variables nor do they rank the variables according to their relative importance in light of multiple interactions among the variables. Hierarchical ranking has become an increasingly useful tool for such analyses as it enables exploration of the relationship among different risk factors, giving a better understanding of their actual importance and the interplay of these factors on infection or reinfection. More specifically, the classification and regression tree (CART) technique is an especially useful decision support tool in this context, with its advantage over the multivariate analysis described in several studies [10,13,14, 15]. CART has been used in ranking determinants of *Taenia solium* infection [10], malaria infection [13], as well as for other diseases [16,17], but not for schistosomiasis infection or reinfection. The objective of the current study was to apply CART analysis to determine the relative importance and relationships among different factors that can predict risk of reinfection with *S*. *mansoni* in school-aged children in Northeastearn Brazil, who had been successfully treated with Praziquantel (PZQ) and then followed for 12 months post-treatment to determine their level of reinfection.

## Methods

### Study area and population

This is a longitudinal study of school-aged children conducted in five municipalities (Itaobim, Jequitinhonha, Joaima, Monte Formoso and Ponto dos Volantes) in the Jequitinhonha Valley in northern Minas Gerais State (MG). This region is known as one of the poorest regions in Brazil, with low social economic indexes. These municipalities together have a population of 76,074 with approximately 36% living in rural areas. The area is endemic for *S*. *mansoni* and is drained by streams and rivers. The main sources of income are subsistence agriculture, animal husbandry, and small-scale commerce.

**Fig 1:**
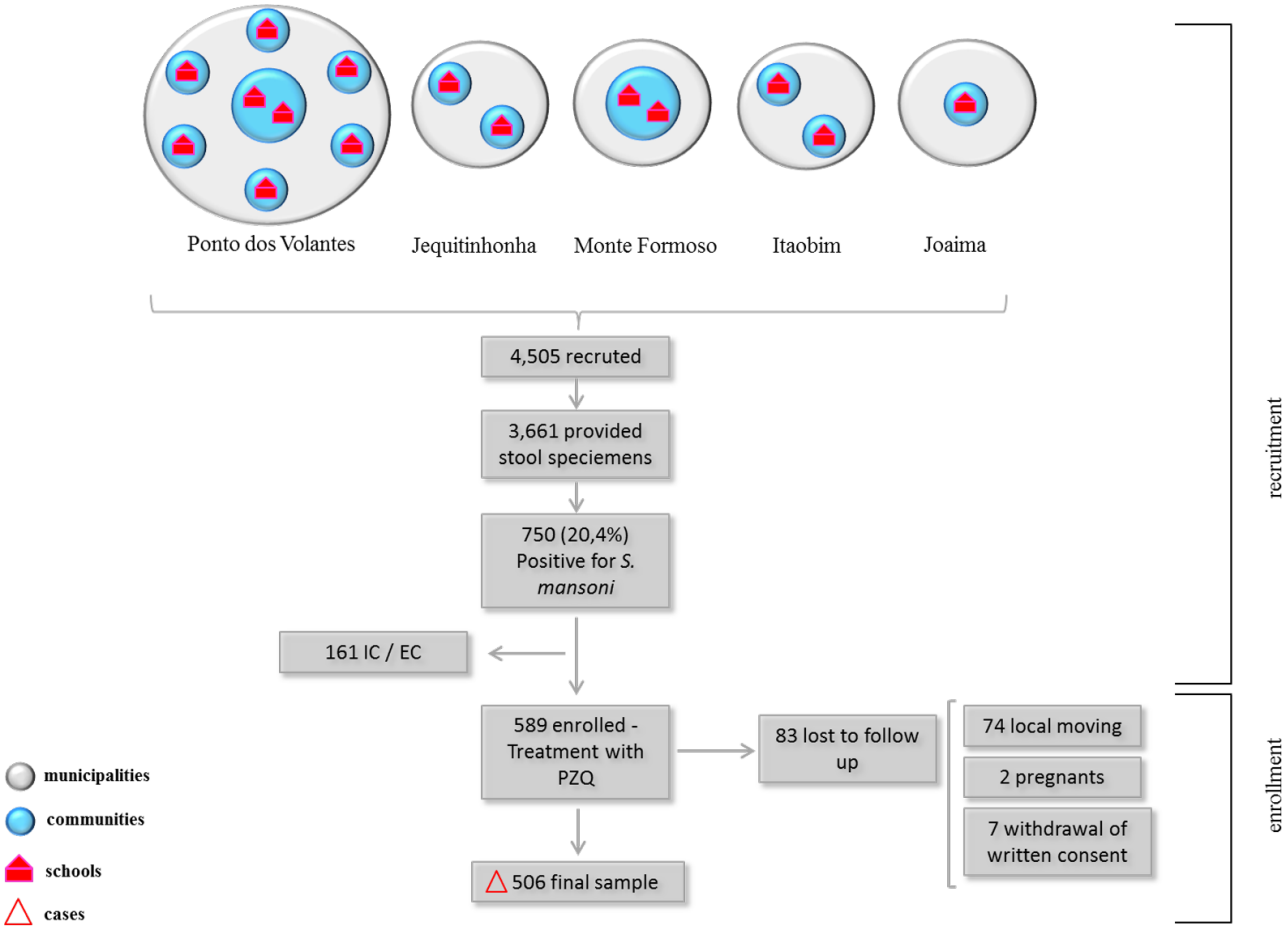
Flow chart representing the recruitment and enrollment phases of the study. Municipalities are represented in grey, communities in blue, schools in red and the final sample represented by triangle. IC / EC: inclusion and exclusion criteria; PZQ: Praziquantel.

**Fig 2:**
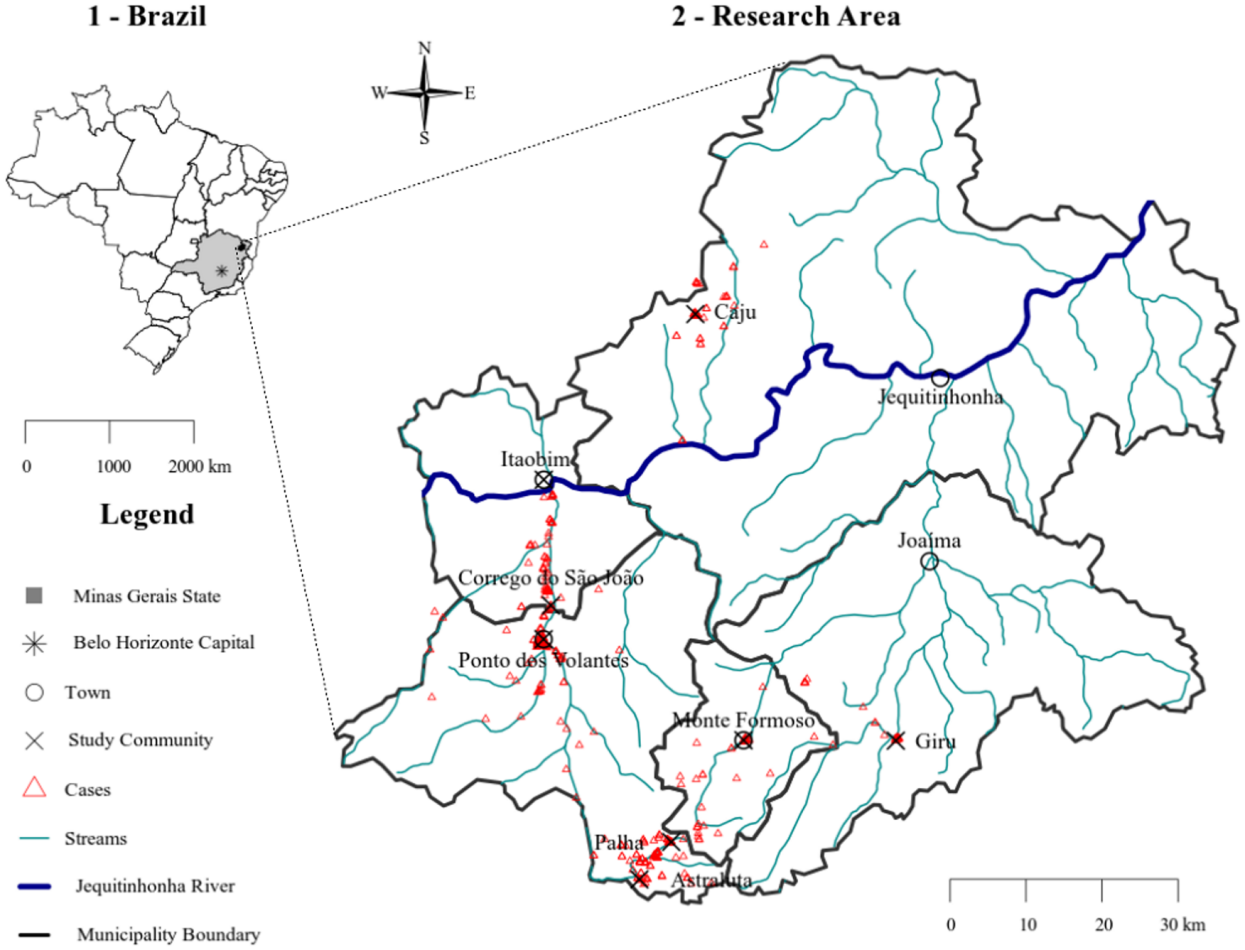
Map showing the distribution of communities in Northeastern, Minas Gerais State Brazil in which participants were enrolled into the longitudinal component of the study. Map created in R statistical software (https://www.r-project.org/) from public data of the Brazilian Institute of Geography and Statistics—IBGE, available at: http://mapas.ibge.gov.br/bases-e-referenciais/bases-cartograficas/malhas-digitais.html and the National Water Agency—ANA (2010) Hydrography 1: 1,000,000 (georeferenced digital base), available at: http://hidroweb.ana.gov.br/HidroWeb.asp?TocItem=4100.

Thirteen communities were indicated by the schistosomiasis control programs of the municipalities as having a higher probability of finding *S*. *mansoni* positive cases. Students from 15 local public schools, eight in Ponto dos Volantes, two in Jequitinhonha, Monte Formoso and Itaobim and a single in Joaíma were chosen for the current study. According to the list of all students from those schools, 4,505 elementary and high school students of both sexes were eligible. These students and their parents or legal guardians were invited by members of the study team to participate in the parasitological screening. After obtaining a written consent, 81.2% (n = 3,661) of the students provided stool specimens, with 20.4% (n = 750) identified as positive for *S*. *mansoni* by conventional microscopy (see Methods below). Parents and legal guardians of the infected children were then visited and informed about the study and their children invited to participate. Only children who were positive for *S*. *mansoni* and between the ages of 6 to 15 years (inclusive) and not pregnant or breastfeeding were enrolled in the study (Fig 1). The final study area was comprised of eight communities as shown in Fig 2.

### Parasitological methods

All study participants received two name-coded, 80-ml plastic containers for the collection of fecal samples. They were instructed to deposit one fecal sample per day in the container and return each sample immediately to collection points, where they were stored at 4°C. Two slides for each stool sample (four slides per individual) were prepared within 24h of collection, using the Kato-Katz fecal thick smear method [18]. For quality control, 10% of the slides were randomly selected and examined by a senior microscopist at the Centro de Pesquisas René Rachou, FIOCRUZ, in Belo Horizonte, MG. The intensity of *S*. *mansoni* infection was estimated from the mean egg count per gram of feces (epg) and categorized into classes of egg counts as recommended by World Health Organization (WHO) [19]: low (1–99 epg), moderate (100–399 epg) and high (≥400 epg). The moderate and high infected groups were combined into a single group for analyses and as such our analyses were based on two groups: low burden (1–99 epg) and moderate to high burden (≥ 100 epg).

Stool samples were collected one week before and one year after successful chemotherapy by PZQ as determined by the absence of eggs in feces by the Kato Katz method. At baseline (D-0), between March and December 2014 in the different study communities, treatment was performed with the standard single oral dose of 60 mg/kg of PZQ administered with direct observation and under medical supervision as recommended by the Brazilian Ministry of Health [20]. Individuals infected with geohelminths were also treated with a single oral dose of 400 mg of albendazole under the same conditions.

Four weeks after PZQ treatment, two stool specimens were collected from each individual on two consecutive days to determine the effectiveness of the treatment. Individuals who remained infected received a single dose of PZQ at 60 mg/kg orally until there was an absence of *S*. *mansoni* eggs four weeks after treatment (successful treatment). One year after the successful treatment (Day 360), two stool specimens were again collected from each individual and examined for *S*. *mansoni* infection, with reinfection defined as the presence of a single *S*. *mansoni* egg by microscopy. Positive cases were retreated with PZQ at 60 mg/kg orally until successful treatment was reached as described above. Reinfection was considered the presence of a single *S*. *mansoni* eggs in the feces by microscopy 360 days post successful treatment with PZQ.

### Socioeconomic and water contact surveys

Questionnaires as previously described [21, 22] were applied to obtain demographic (age, gender, place of residence, education of household head), socioeconomic (income, wealth, occupation, quality of housing, presence and type of latrine, number of persons per room and water supply) and water contact behavior information for all participants at baseline. Data was collected by graduate students, specifically trained and supervised by the team leader. Parents answered the questions on socioeconomic status and water contact for children less than 10 years of age.

The socioeconomic information was scored to determine the socioeconomic status (SES) of families using the method of Gwatkin et al. [23]. This instrument considers household appliances, car ownership, type of water used, etc. as a gauge of a household socioeconomic status rather than monthly income alone. The asset questionnaire is binary (“yes” or “no”) and is summarized and scored. The sum of scores allows the separation of families into wealth quintiles with cut-off points as follows: low (less than -0.66129); second (-0.66129 to 0.02018); third (0.02018 to 0.51776); fourth (0.51776 to 1.00965) and high (above 1.00965). The lowest quintile was designated as “extremely poor”, the second quintile as “very poor”, the third quintile as “poor”, the fourth quintile as “medium”, and the fifth as “high” socioeconomic status. In this study, all families were ranked in the three lowest quintiles.

All participants were asked about the type and frequency of their weekly contact with potentially *S*. *mansoni* infected water sources for domestic, recreational, hygienic, and occupational purposes. Contact with the surface of water bodies, including streams, canals, springs, and ponds, was also included because of the widespread distribution of the intermediate host *Biomphalaria glabrata* in the study area. The weekly frequency of water contact data was used to calculate the exposure index or Total Body Minutes (TBM) developed by Kloos et al. [24]. All predictor variables used in the CART analysis are described in Table 1.

**Table 1:** Predictor variables in the CART analysis for risk of *S*. *mansoni* reinfection.

Variable classes	Predictor variables
**Host characteristics**	Age
	Gender
	Education of household head
	Intensity of infection
	Socioeconomic level
**Household characteristics**	Locality (rural or urban)
	Presence of latrine
	Type of latrine
	Type of walls
	Type of floor
	Treated water supply
**Water contact**	Total TBM
	Washing clothes
	Washing utensils
	Washing animals
	Washing parts of the body
	Bathing
	Swimming
	Playing
	Fishing
	Watering plants
	Irrigation
	Collecting sand at stream
	Fetching stream water
	Crossing streams
	Cleaning streams

### Statistical analysis

All data were double entered by two individuals into Excel spreadsheets (Microsoft Office Excel 2007, Microsoft Corp., Redmond, WA). Then the two data sets were crosschecked by a third individual and merged into a single data set for analysis. If a discrepancy was identified, data were again checked and if necessary return to subject. Chi-square test was used to test for difference in proportions with a statistical significance at a p<0.05. The variables with p-values <0.20 in the univariate analysis were selected. Subsequently multivariate adjusted analysis and a Poisson regression with robust variances were used. The magnitude of associations was estimated by relative risk (RR), assuming a 95% confidence interval (CI) as precision measurement. Variables remaining statistically significant below 5% were retained in the final model.

Quality evaluation of the model was carried out by calculating its coefficient of R2 determination and by goodness-of-fit test. Co-linearity and interaction between the variables in the final model were also tested. The software Statistical Package for Social Sciences (SPSS Inc., Chicago, USA) version 19.0 and the Statistical Software for Professionals (STATA) Version 12 were used for data analysis.

The Classification and Regression Tree (CART) model was used to show the relationship among risk factors that lead to a higher risk of reinfection and their hierarchical classification. The classification model was developed using the method first introduced by Breiman [25], known as Decision Trees (S1 Box). CART is a non-linear and non-parametric model that is fitted by binary recursive partitioning of multidimensional covariate space [25]. Classification trees are used to analyze categorical health outcomes, while regression trees serve to assess continuous health outcomes [15]. See S1 Box for the details of CART analysis.

### Ethical approval

Ethical approval was obtained by the Brazilian National Ethics Committee (CONEP-Number 531.282). Prior to the commencement of the study, a written consent was obtained from all parents and legal guardians of participants and informed assent obtained from students aged ≥ 7 years.

## Results

### Study sample

A total of 589 students aged 6 to 15 years old (inclusive) from 10 schools in eight communities were included in the study sample. Eighty-three students were lost to follow-up after one year: 74 (89.1%) moved to other areas, 2 (2.4%) got pregnant and 7 (8.4%) withdrawal of written consent. The final sample for the study consisted of 506 individuals infected with *S*. *mansoni*. The population of the study are mostly from poor families, with 82.6% of them receiving government assistance through the “Bolsa Família Program”, a social welfare initiative of federal assistance. They typically live in houses with no sewage system, with only 57.1% of the households having treated water.

### Risk factor analysis

A total of 506 children and adolescents infected with *S*. *mansoni* participated in the study, and 111 of the 506 were reinfected one year after treatment. The intensity of infection with *S*. *mansoni* before treatment (*baseline)* was determined by geometric mean eggs per gram of feces (epg) to be 79.26 (95% CI, 23.90, 134.61). Treatment with PZQ resulted in substantial reductions both on the number of individuals reinfected with *S*. *mansoni* and a reduction in their egg counts (epg) 12 months after successful treatment (Study Day 360). The reinfection rate one year after treatment was 21.9% (n = 111) and the intensity of infection was 20.49 epg (95% CI, 2.85, 38.13). Table 2 shows that reinfected individuals were mostly males (n = 65, 58.6%), aged from 11 to 15 years (50.5%) (n = 56), living in rural areas (n = 72, 64.9%), with a moderate to high intensity of infection of (n = 71, 64%),and a household head with 1 to 4 years of schooling (n = 55, 49.5%) and classified as very poor household (n = 61, 55%) (Table 2). When comparing “reinfected” individuals with those who were not reinfected, we observed a significant difference (p <0.001) in intensity of infection since most of the reinfected individuals had moderate/high intensity of infection.

**Table 2:** Baseline and reinfection demographic and socioeconomic factors and intensity of infection of reinfected and non-reinfected students.

	Study Sample (n = 506)		Reinfected (n = 111)		Not reinfected (n = 395)		*p*
Variables	n	%	n	%	n	%	
**Gender**							
Male	297	58.7	65	58.6	232	58.7	0.974
Female	209	41.3	46	41.4	163	41.3	
**Age (years)**							
5–10	225	44.5	56	50.5	170	43	0.223
11–15	281	55.5	55	49.5	225	57	
**Local**							
Rural	318	62.8	72	64.9	246	62.3	0.618
Urban	188	37.2	39	35.1	149	37.7	
**Intensity of infection**							
Moderate-high	209	41.3	71	64.0	138	34.9	**<0.001**
Low	297	58.7	40	36.0	257	65.1	
**Education (Household head)**							
None	136	26.9	30	27.0	106	26.8	
Grades 1–4	216	42.7	55	49.5	161	40.8	0.145
Grades > 4	154	30.4	26	23.4	128	32.4	
Economic classification*							
Extremely poor	184	36.4	39	35.1	145	36.7	
Very poor	240	47.4	61	55.0	179	45.3	0.073
Poor	82	16.2	11	9.9	71	18	

*Note that no household reached the “medium” or “high” economic strata.

A Poisson regression model adjusted for demographic, socioeconomic, water contact and intensity of infection before treatment was performed. The results showed that only the variable “intensity of infection” at baseline remained independently associated with reinfection one year after treatment. Individuals who had moderate/heavy intensity of infection before treatment had a higher risk of reinfection for *S*. *mansoni* (RR = 2.52; 95% CI, 1.78, 3.56.

### Classification and regression trees (CART)

According to the overall discriminatory power in the analysis (Table 3), the intensity of infection at baseline was the most important factor (100.0) in predicting the risk of reinfection with *S*. *mansoni*. This was followed by Total Body Minutes in potentially *S*. *mansoni* contaminated water (94.9) and by washing parts of the body in potentially *S*. *mansoni* contaminated water (75.3). Other factors are shown in Table 3.

**Table 3:** Ranking of reinfection risk factors by overall discriminatory power.

Variable	Power
Baseline intensity of infection	100.0
Total TBM	94.9
Washing parts of the body	75.3
Type of latrine	36.0
Type of floor	26.3
Socioeconomic level	25.6
Playing	25.5
Presence of latrine	25.0
Age	23.9
Washing animals	22.2
Bathing	21.5
Swimming	16.8
Education of household head	14.1
Fetching water	13.4
Gender	12.7
Crossing streams	10.6
Fishing	10.1
Chemically treated water	7.5
Locality	7.3
Collecting sand at streams	6.7
Type of walls	3.1
Washing clothes	0.0
Washing utensils	0.0
Watering plants	0.0
Irrigation	0.0
Cleaning stream	0.0

**Fig 3:**
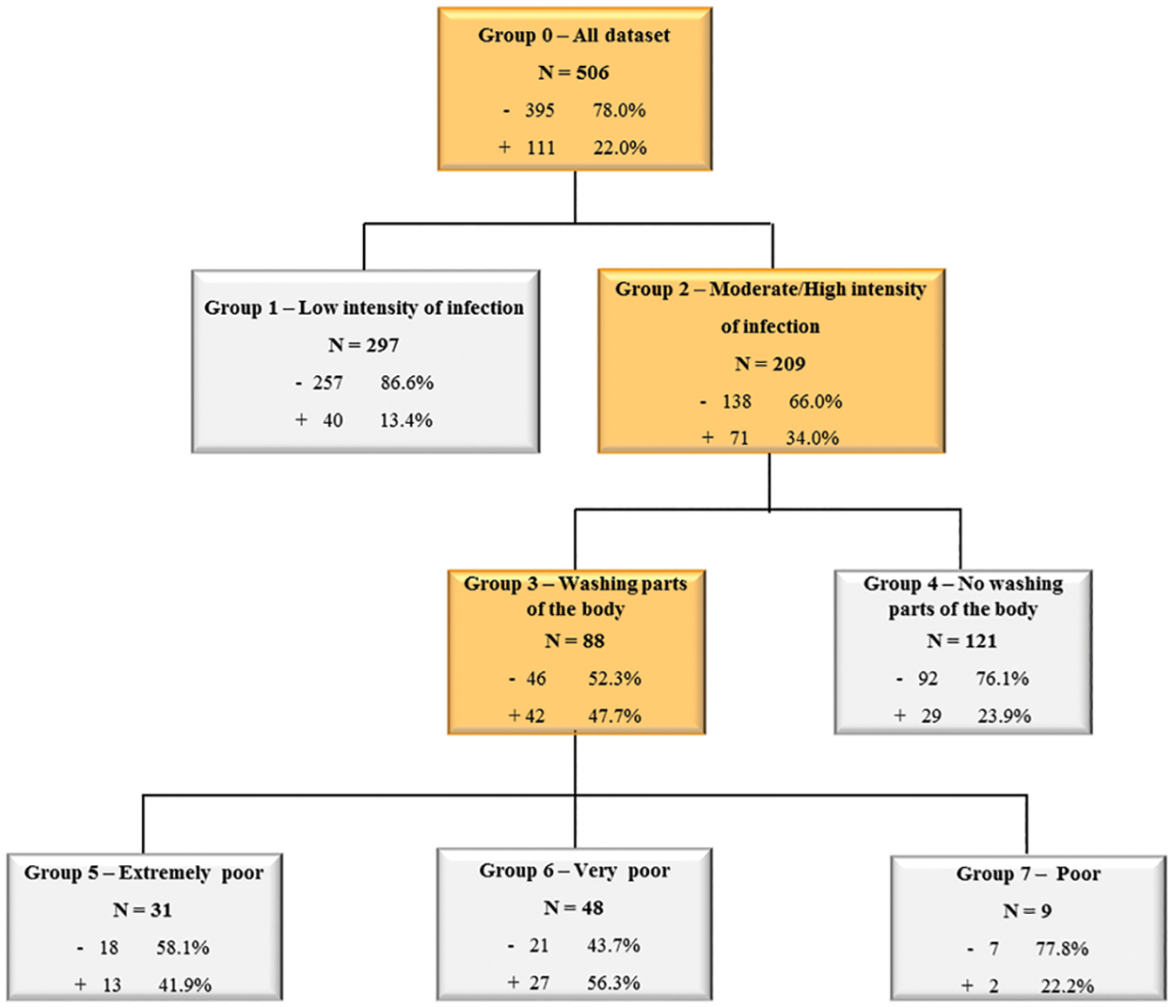
Classification tree of the risk factors for reinfection. The internal nodes are highlighted in orange. The plus sign (+) represents positivity for reinfection and the minus sign (-) represents lack of reinfection.

The classification tree (Fig 3) showed that at the root, 111 (22%) of the 506 individuals were reinfected with *S*. *mansoni*. The intensity of infection (expressed as an EPG) at baseline was the first “splitter”, with individuals having a moderate to heavy intensity of infection (n = 209) at baseline having a greater risk of becoming reinfected (34.0%) than individuals with a low intensity of infection at baseline (13.4%). Moreover, in the former group (second splitter), reinfection was higher among moderate to heavily infected individuals who had a higher mean TBM for washing parts of the body (47.7%) than for those with lower mean TBM (23.9%). For those with higher mean TBM for washing parts of the body, socioeconomic classification was the best discriminator of their placement in this group. Reinfection was higher in the very poor socioeconomic strata (56.3%) and in the extremely poor strata (41.9%) if compared with reinfection in the “poor” socioeconomic strata (22.2%) (Fig 3).

CART analysis showed that individuals who had a low pre-treatment intensity of infection had substantially lower risk of reinfection than the rest of the sample (13.4% versus 22.0%). Among those with moderate-high intensity of infection, CART analysis split them further into homogeneous reinfection risk groups. Groups 4 and 7 are nearly equal with the rest of the sample and are composed of participants who either do not wash parts of their bodies in potentially *S*. *mansoni* contaminated water nor wash parts of their bodies in potentially *S*. *mansoni* contaminated water, but are in the “poor” socioeconomic strata. The two remaining groups, composed by those who wash parts of their body in potentially *S*. *mansoni* contaminated water and in the “extremely poor” socioeconomic strata have a higher risk of reinfection than the overall sample (56.3% and 41.9, respectively).

**Fig 4:**
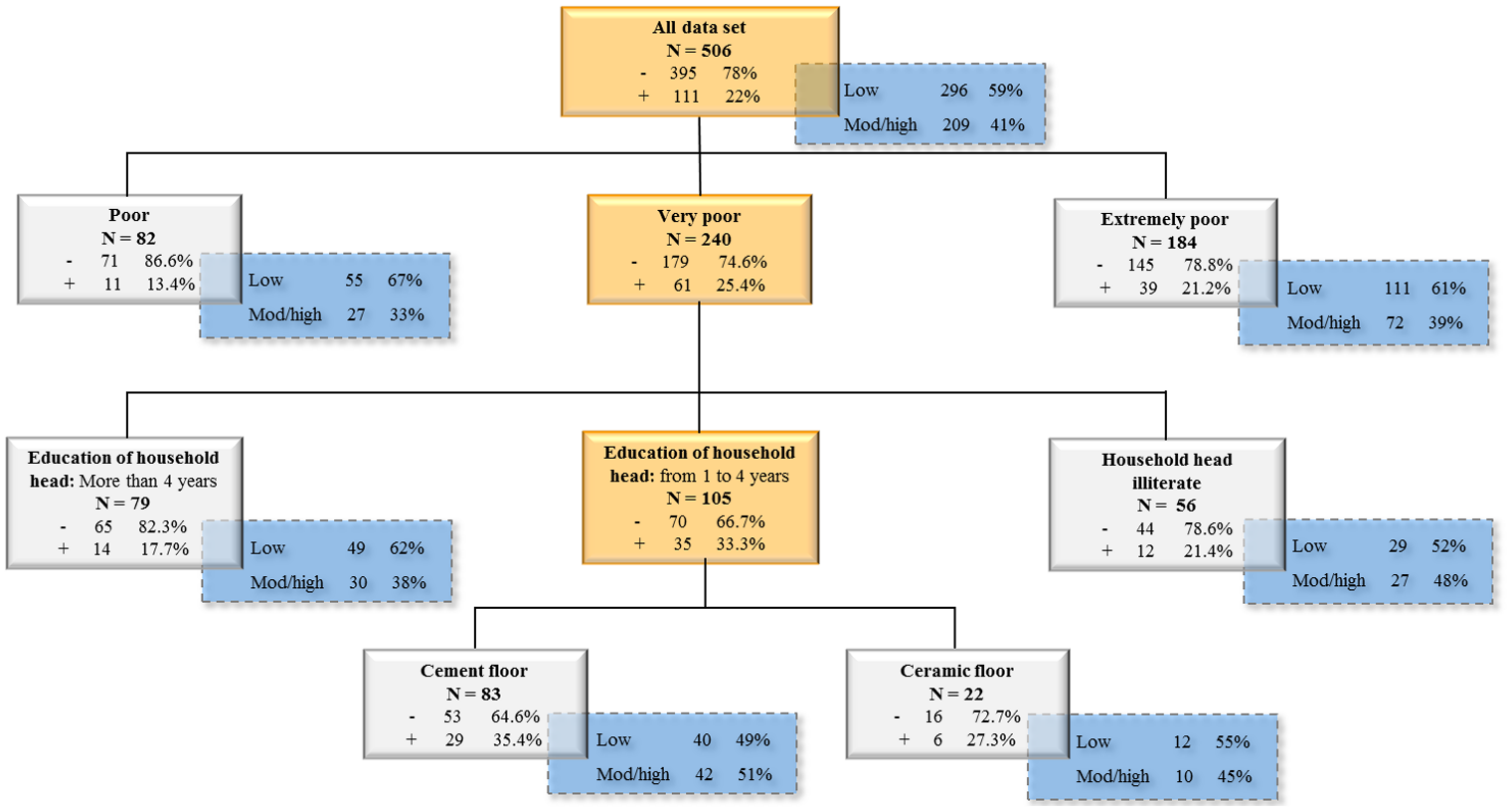
Classification tree of the risk factors for reinfection excluding the variable intensity of infection at baseline. The internal nodes are highlighted in orange. The plus sign (+) represents positivity for reinfection and the minus sign (-) represents no reinfection. The crosshatched boxes (blue) show the percentages of those with low and moderate/high intensity of infection in each final node.

It is possible that the variable “intensity of infection” was affected by the other variables in CART, making it the causal factor that best describes reinfection, since it is the first splitter of the tree (Fig 3) and with the greatest discriminatory power (Table 3). Therefore, the variable intensity of infection at baseline was removed, and CART analysis runned on weka, generating a different classification tree. With the resulting tree, the rate of low and moderate/high intensity of infection in each final node was manually calculated and put together to be able to visualize the behavior of both variables: reinfection and intensity of infection. The resulting analysis showed other variables not present in the first tree (Fig 3), but important risk factors for reinfection (Fig 4). The tree showed that in the first splitter the very poor and the extremely poor individuals had higher reinfection rates (25.4% and 21.2% respectively) than the poor individuals (13.4%). In the former group, reinfection was higher among children whose head of household had 1–4 years of education (33.3%) or were illiterate (21.4%) in comparison with literate heads of households (17.7%). For those with head of household with a low level of education, the type of floor was the best discriminator of reinfection. Over one third of the 83 (35.4%) reinfected individuals lived in houses with cement floor, but only 27.3% of the 22 individuals living in houses with ceramic tile floors were reinfected.

Other factors also significantly influenced reinfection. Using the resulting tree structure of re-run CART without intensity of infection, the percentages of low and moderate/high intensity of infection category were manually calculated for each leaf of the tree and put together (Fig 4). The effects of two variables (education of household head and type of floor) were hidden by the presence of the initial intensity of infection as a possible explanatory variable. Indeed, these variables may also explain the initial intensity of infection. The percentages of those with low and moderate/high intensity of infection were calculated and results are shown in a crosshatched box attached to each final node in Fig 4. We observed that, by considering the final groups, the percentage of reinfection is positively correlated with the percentage of moderate/high intensity of infection. For example, the individuals classified as poor had a lower risk of reinfection (13.4% in the group versus 22% in the entire sample) and they too have a lower risk of moderate/high infection intensity in the crosshatched box (33% in the group versus 41% in the entire sample). In this study the two trees analysis indicate that intensity of infection, water contact, poverty and education are important risk factors for reinfection in the endemic areas studied.

## Discussion

Schistosomiasis is a disease with complex transmission dynamics that require sophisticated statistical analyses at the local level to capture the relationships among various risk factors and the hierarchical importance of each risk factor on infection and reinfection. Indeed, surveys in *S*. *mansoni* endemic areas often generate large and complex data sets, with a broad range of variables, requiring the application of appropriate statistical analyses. CART is a statistical method that allows the hierarchical analyses of such complex data sets and generates a classification tree, that uses all possible combinations to show relationships between variables and rank the potential factors related [15], enabling the application of these results to control and possibly eliminate *S*. *mansoni* in endemic areas. In CART analysis, the discriminatory power of the model provides a ranking based on the overall contribution of each variable in the construction of the tree. This hierarchical ranking indicates the importance of each independent variable as a predictor, while the tree allows the exploration of relationships between variables and their relative importance [15]. In this study, some risk factors appear in the first tree from the root (Fig 3) in a different ranking than their hierarchical importance shown in the discriminatory power in Table 3. This is due to the tree being able to show the splitter variables that increase the differentiation between groups (reinfected and non-reinfected), conditioned on the values of previous variables. This creates a set of hierarchically nested groups showing a highly homogeneous reinfection behavior. In general, the high risk factors determined by CART and the most important variables presented in the discriminatory power table (Table 3) are quite similar. The arrangement and sequence of risk factors in the tree shows the interaction and relationship among these variables that result in reinfection.

In the current study, the final regression model showed that the moderate/high intensity of infection at baseline was the only risk factor for reinfection. The CART analysis confirms the final Poisson regression model, in addition, identified other important risk factors in predicting reinfection according to their importance, showing the advantages of this method. CART has the advantage of dealing with multi-collinearity, multi-level interactions and can identify risk factors according to their importance, different from multinomial models such as logistic regression (15).

As mentioned, a critical risk factor for reinfection was intensity of infection at baseline. This may be related to the fact that in our studies, we focused on a population of young individuals that are constantly exposed to contaminated water. In this context, it is possible that an increase on reinfection levels may be a consequence of cumulative infections due to the lack of an effective immune response that allows for the killing of all the incoming parasites leaving some to develop to the adult stage. Therefore, it is possible that the observed infection levels, after treatment, may be due to the, yet, ineffective immune response of the young individuals to new incoming infection in these endemic areas [26, 27].

Heavily infected individuals have most of the clinical consequences of schistosomiasis and are the major source of infection for the rest of the community [19]. Anemia is more common in children with moderate to heavy *S*. *mansoni* infection and the prevalence of hepatomegaly increases in heavily infected children [28]. High and moderate intensity of infection are also associated with increased risk of morbidity in schistosomiasis such as liver fibrosis, and genitourinary and gastrointestinal abnormalities [28–33].

However, there is increasing evidence demonstrating that light infections are epidemiologically important since they contribute significantly to maintain the transmission cycle [34].

The CART analysis also showed that besides moderate to heavy pre-treatment infection intensities, other risk factors were important in predicting reinfection. Several water contact activities, specifically washing parts of the body, were identified as important risk factors for reinfection as shown by the ranking of its discriminatory power and its appearance as a main “splitter” in the first tree (Fig 3). These activities are often associated with a short duration and medium degree of body exposure compared to other water contact activities such as bathing, swimming, playing and washing clothes and utensils.

Studies in Brazil evaluating the risks of infection by Matoso et al. [5] showed an increased risk in children and young adults who fished and crossed streams. Similar observations with schoolchildren have been shown by Massara et al. [35], who observed that activities with both high and low water contact exposure (e.g., bathing, fishing, crossing streams, working in agriculture, watering plants and working in sand extraction) were significantly associated with infection. Studies with school age children in Sub Saharan Africa were done mostly with *S*. *hematobium* and showed controversial results. Satayathum et al. [36] found that the frequency of water contact was not a predictor of risk for *S*. *hematobium* infection or reinfection. On the other hand, Rudge et al. [37] observed that particular activities associated with a long duration and a high degree of body surface exposure (e.g., swimming, bathing and fishing) were strongly associated with *S*. *hematobium* infection. These results suggest that the frequency, duration, and the percentage of body exposure in water do not need to be exceptionally high to result in high infection rates. In our CART analysis, washing parts of the body was observed to be an important risk factor for reinfection in children with.moderate/high intensity of infection.

Another important risk factor observed was SES classification. This factor was detected both on the first and second tree of the CART analysis. In the second tree, two other socioeconomic indicators (education of household head of family and type of floor) also showed that lower SES classification was an important determinant for reinfection, regardless of infection intensity at baseline. One possible explanation is that lower SES classification reduces access to safe water supplies and sanitation as well as to health care, thus increasing the risk of reinfection and morbidity [31]. It is important to note that the great majority of schistosomiasis infected people live in marginalized, low-income, resource-limited regions with inadequate sanitation [38, 39]. This situation reaffirms what is known about the relationship between social resources, economic status, and schistosomiasis infection at the community and the household level, particularly among school children in low-income localities as shown in a meta analysis by Houweling et al. [40]. Brazil has numerous low-income places, where schistosomiasis has long be in transmission, further supporting our findings [41, 42].

In summary, the most important risk factors for reinfection among school age children as determined by our CART analysis were intensity of infection at baseline, lower SES classification, and the head of the households level of education, all of which are useful and easily obtained indicators of risk of reinfection. As such, efforts to control schistosomiasis reinfection can be stratified to target the treatment and monitoring of such at risk children. For example, when baseline classification has been determined, school aged children with moderate to high intensity of infection may be a priority for treatment, as this group has a greater likelihood of becoming reinfected than other children in the community. In the absence of classification, surrogate indicators of reinfection such as low household economy or the low level of education of the head of the household may enabled targeted treatment or monitoring. In this case, the data generated by census (such as by the Instituto Brasileiro de Geografia e Estatística-IBGE) could contribute to screening the regions where income and education are low and through socioeconomics questionnaires would be identified target families for treatment. This analytic approach may help to guide the Brazilian government's schistosomiasis control program action proposal of which calls for treating communities in high-risk areas to reduce disease transmission and morbidity. The ranking of independent factors may help prioritize prevention and control efforts focusing on specific factors and thus facilitate planning and organizing efficient and cost-effective strategies in the prevention and control of schistosomiasis.

## Supporting information

S1 Box Cart for field analyses. (DOC)

S1 File Database. (SAV)
