# Supplementary material for: Schistosoma mansoni reinfection: Analysis of risk factors by classification and regression tree (CART) modeling
Source: PLoS One. 2017 Aug 16;12(8):e0182197. doi: 10.1371/journal.pone.0182197 (PMC5558968; doi:10.1371/journal.pone.0182197)
Supplement: S1 Box — (DOC) [file pone.0182197.s001.doc]

| **S1 Box. CART FOR FIELD ANALYSES** |
| --- |
| CART analysis was used to successively split data from a large number of independent variables into increasingly homogeneous subsets until it is stratified to meet a specified criterion. On each division performed, the data are separated in accordance with common features to arrive at indivisible points that represent the classes for statistical analysis, based on recursive partitioning of the data, in which a predictive model is built in the form of a tree. The method works, starting at the root node containing all individuals in the data set in the tree, then built recursively. The root is then split into two child nodes and then, into two or more groups. This process is repeated recursively for each group, until a stopping criterion is achieved or the data cannot be further split. Following the path from the root to a leaf node, the successive splits delimit a partition of the data, which is classified with the label present in the leaf node. In other words, the objective of each split is to maximize the homogeneity between patients classified in each child node and to maximize the heterogeneity between these two nodes. CART presents its results in the form of a decision tree, a different approach than the better known parametric techniques [15]. We selected CART as an appropriate alternative predictive/exploratory analytic tool, given its simplicity and versatility. In addition, CART is characterized by its flexibility, without any restrictions about the nature or distribution of variables and its simplicity in model construction and results interpretation [1]. After the tree is constructed, the algorithm employs a pruning method to avoid overfitting. We used the C4.5 algorithm [2], available in Weka 3.7, an open-source data mining software [3]. This algorithm uses information gain (a metric based on the concept of entropy from information theory) to select the variable at each node that is used to split the data (Table 1). For this model, a 10-fold cross validation was performed as the method for testing the predictive capacity of the trees. A minimum of 30 samples per terminal node was selected to avoid too many splits, with few observations, that are difficult to explain. The CART also provides a score indicating the importance of the different variables. This discriminatory power is reported relative to the most important variable (which is given a score of 100). |

**References**

1. Merow C, Smith MJ, Edwards TC, Guisan A, Mcmahon SM, Normand S, et al. What do we gain from simplicity versus complexity in species distribution models? Ecography. 2014;37(12):1267–81.

2. Quinlan JR. Induction of Decision Trees Mach Learn. 1986;1(1):81–106.

3. Hall M, Frank E, Holmes G, Pfahringer B, Reutemann P, Witten IH. The WEKA data mining software. ACM SIGKDD Explor Newsl. 2009;11(1):10.
